# Clinical and laboratorial features of spontaneous bacterial peritonitis in southern Brazil

**DOI:** 10.1590/S0100-7203201400040004

**Published:** 2014-05-20

**Authors:** Gabriela Bicca Thiele, Otávio Marcos da Silva, Leonardo Fayad, César Lazzarotto, Mariana do Amaral Ferreira, Maíra Luciana Marconcini, Esther Buzaglo Dantas-Corrêa, Leonardo de Lucca Schiavon, Janaína Luz Narciso-Schiavon

**Affiliations:** I Medical Student. Universidade Federal de Santa Catarina (UFSC), Florianópolis, Santa Catarina, Brazil; II MD. Resident in Gastroenterology, Núcleo de Estudos em Gastroenterologia e Hepatologia (NEGH), Universidade Federal de Santa Catarina (UFSC), Florianópolis, Santa Catarina, Brazil; III MD, MSc. Resident in Gastroenterology, Núcleo de Estudos em Gastroenterologia e Hepatologia (NEGH), Universidade Federal de Santa Catarina (UFSC), Florianópolis, Santa Catarina, Brazil; IV MD, PhD. Adjunct Professor in Gastroenterology, Núcleo de Estudos em Gastroenterologia e Hepatologia (NEGH), Universidade Federal de Santa Catarina (UFSC), Florianópolis, Santa Catarina, Brazil

**Keywords:** Liver cirrhosis, Ascites, Peritonitis, Ascitic fluid, Paracentesis, Cirrose hepática, Ascite, Peritonite, Líquido ascítico, Paracentese

## Abstract

**CONTEXT AND OBJECTIVE:**

Spontaneous bacterial peritonitis (SBP) is a severe complication that occurs in 8-27% of hospitalized patients with liver cirrhosis and ascites, with high mortality rates. This study aimed to identify the clinical characteristics associated with SBP.

**DESIGN AND SETTING:**

Cross-sectional study, conducted in a public university.

**METHODS:**

The study consecutively included individuals with liver cirrhosis and ascites between September 2009 and March 2012. Forty-five patients were included: mean age 53.2 ± 12.3 years, 82.2% male, 73.8% Caucasian, mean Model of End-stage Liver Disease (MELD) score of 19.5 ± 7.2, and 33.3% with SBP. The subjects were divided into two groups: SBP and controls.

**RESULTS:**

Comparison between individuals with SBP and controls showed that those with SBP had lower mean prothrombin activity time (36.1 ± 16.0% versus 47.1 ± 17.2%; P = 0.044) and lower median serum-ascites albumin gradient (SAAG) (1.2 versus 1.7, P = 0.045). There was a tendency towards higher mean MELD in the SBP group, not significant (22.2 ± 7.6 versus 17.9 ± 6.7; P = 0.067). There was a strong positive correlation between the neutrophil count in ascitic fluid and serum leukocyte count (r = 0.501; P = 0.001) and a negative correlation between the neutrophil count in ascitic fluid with prothrombin activity time (r = -0.385; P = 0.011).

**CONCLUSION:**

A few characteristics are associated with the presence of SBP, especially liver dysfunction, SAAG and peripheral leukocytosis.

## Abstract (pt)

**CONTEXTO E OBJETIVO:**

Peritonite bacteriana espontânea (PBE) é uma complicação grave que ocorre em 8-27% dos pacientes hospitalizados com cirrose hepática e ascite, e apresenta altas taxas de mortalidade. O objetivo deste estudo é identificar as características clínicas associadas à PBE.

**TIPO DE ESTUDO E LOCAL:**

Estudo transversal, conduzido em uma universidade pública.

**MÉTODOS:**

O estudo incluiu, consecutivamente, indivíduos com cirrose hepática e ascite entre setembro 2009 e março 2012. Foram incluídos 45 indivíduos com média de idade de 53,2 ± 12,3 anos, sendo 82,2% homens, 73,8% brancos, com MELD (Modelo para Doença Hepática Terminal) de 19,5 ± 7,2, e 33,3% com PBE. Os indivíduos foram divididos em dois grupos: PBE e controles.

**RESULTADOS:**

Quando se compararam os indivíduos com PBE aos controles, observou-se menor média de tempo de atividade da protrombina (TAP; 36,1 ± 16,0% *versus* 47,1 ± 17,2%; P = 0,044) e menor mediana de gradiente albumina soro-ascite (GASA; 1,2 *versus* 1,7; P = 0,045). Houve tendência do grupo com PBE de apresentar maior média de MELD, sem significância estatística (22,2 ± 7,6 *versus* 17,9 ± 6,7; P = 0,067). Foi observada forte correlação positiva entre neutrófilos do líquido ascítico e contagem sérica de leucócitos (r = 0,501; P = 0,001) e correlação negativa de neutrófilos do líquido ascítico com TAP (r = -0,385; P = 0,011).

**CONCLUSÃO:**

Poucas características estão associadas à presença de PBE, em especial a disfunção hepática, o GASA e a leucocitose periférica.

## INTRODUCTION

Spontaneous bacterial peritonitis (SBP) is found in 8% to 27% of the patients hospitalized with liver cirrhosis and ascites, and presents high rates of intra-hospital mortality, of between 20 and 40%^1^ ^-^ ^3^ Studies have suggested that the recurrence rates are high: more than 70% within one year.^4.5^

In the great majority of the cases, the bacteria that cause SBP come from the digestive tract. Extra-intestinal bacteria, such as those from the respiratory and urogenital tracts or the skin, are much less frequent. Catheters and other equipment used during invasive procedures represent another possible source of infection. Currently, the most accepted hypothesis regarding the pathogenesis of SBP consists of a bacteremia episode during fluid exchange between the peritoneal and intravascular cavities, with consequent infection of the ascitic fluid.^6^ Aerobic Gram-negative bacteria (most frequently *Escherichia coli*) are considered responsible for the majority of SBP cases, through translocation of the intestinal lumen.^5^ ^,^ ^7^

In fact, only a few patients with SBP present typical symptoms suggestive of peritoneal infection, such as fever, abdominal pain and peripheral leukocytosis. SBP is most frequently suspected when the patient develops signs of hepatic encephalopathy, increased abdominal volume or renal dysfunction, without any apparent precipitating factor. In addition to this, in a significant proportion of the cases, SBP may be completely asymptomatic and the diagnosis can only be made by analyzing the paracentesis results.^8^ If ascitic fluid infection is suspected (fever, abdominal pain, unexplained encephalopathy, azotemia, acidosis, hypotension or hypothermia), total and differential cellularity tests and ascitic fluid culturing need to be requested, with inoculation of the material into blood culturing bottles at the bedside.^9^ ^,^ ^10^

The diagnosis for SBP consists of polymorphonuclear (PMN) counts ≥ 250 cells/mm³ and a positive ascitic fluid culture, without any evidence of external or intra-abdominal infectious source. Neutrophilic ascites is defined by negative cultures and also by PMN counts in the ascitic fluid higher than 250 cells/mm³. Presence of a positive ascitic fluid culture with neutrophil count less than 250 cells/mm³ is diagnosed as bacterascites, and management of this condition varies from patient to patient.^11^

Given the scarcity of studies assessing characteristics of patients with SBP in Brazil, and also the regional differences that exist, there is a need to evaluate this infection in our setting. Through observing its behavior, new strategies aiming towards diagnostic improvement can be sought. 

## OBJECTIVE

The main objective of this study was to identify the characteristics associated with presence of SBP in individuals with decompensated liver cirrhosis with ascites. We also aimed to describe the clinical profile of individuals with SBP.

## METHODS

This cross-sectional analytical study was conducted by reviewing the medical records of individuals with decompensated liver cirrhosis with ascites who were admitted to the gastroenterology ward of the "Polydoro Ernani de São Thiago" university hospital of the Federal University of Santa Catarina (Universidade Federal de Santa Catarina, UFSC), between September 2009 and March 2012. Over the same period, we evaluated the results from ascitic fluid cultures made in the university hospital laboratory of UFSC, for inclusion in the study. Among these, after analysis of the medical files, we excluded the patients who did not have cirrhosis (patients with ascites from other causes) and also those with insufficient clinical and laboratory data registered in their medical records. 

The study protocol conformed to the ethical guidelines of the 1975 Helsinki Declaration and had been approved by our institutional review board under the number 885/10.

Patients were interviewed regarding their demographic and clinical characteristics as stated below. Additional clinical and laboratory variables relating to all the individuals from medical charts. The following clinical variables were studied: age; gender; skin color; SBP (which was defined as a neutrophil count in ascitic fluid higher than 250 cells/mm³ and/or a positive culture); jaundice; hepatic encephalopathy; upper gastrointestinal bleeding (UGIB) during hospitalization; ascitic fluid cultures; maximum axillary temperature; abdominal pain; diarrhea; comorbidities: diabetes mellitus (DM), systemic arterial hypertension (SAH), dyslipidemia and HIV; etiology of the cirrhosis: alcohol, hepatitis C or hepatitis B; and duration of prophylactic antibiotics for paracentesis. Among the laboratory variables, the following were evaluated: neutrophil count in ascitic fluid; prothrombin activity (PA); serum-ascites albumin gradient (SAAG); hemoglobin; leukocytes; platelet count; aspartate aminotransferase (AST), alanine aminotransferase (ALT), alkaline phosphatase (ALP) and gamma glutamyl transferase (GGT); albumin; total and direct bilirubin; creatinine; sodium; glucose; total protein in the ascitic fluid; and albumin in the ascitic fluid. The results from the liver biochemical tests (AST, ALT, ALP and GGT) were expressed as the number of times the upper limit of normal (xULN). The other laboratory variables were expressed as absolute values. The bilirubin tests, international normalized ratio (INR) and creatinine were used for calculating MELD (Model of End-stage Liver Disease).^12^ For analysis purposes, the patients were divided into two chronological groups: SBP and controls (cirrhotic individuals with ascites but no evidence of infection).

### Statistical analysis

Continuous variables were compared using Student's t test, or the Mann-Whitney test when appropriate. Categorical variables were compared using the chi-square test or Fisher's exact test. P values less than 0.05 were considered to be statistically significant. The correlation between the neutrophil count in the ascitic fluid and laboratory variables was assessed using Pearson's correlation coefficient. All tests were performed using the Statistical Package for the Social Science (SPSS, Chicago, Illinois, United States), version 17.0. 

## RESULTS

**Figure 1:**
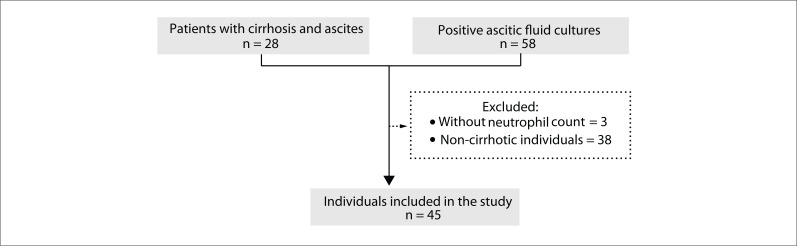
Flowchart of potential candidates for inclusion in the study, application of exclusion criteria and individuals included.

From September 2009 to March 2012, 86 patients were evaluated for inclusion in the study because they presented decompensated cirrhosis with ascites and/or positive results from ascitic fluid cultures in the laboratory. Three individuals were excluded from the study because no neutrophil count in the ascitic fluid was available, along with another 38 patients who did not present cirrhosis (Figure 1). 

We included 45 patients with decompensated cirrhosis with ascites in the study, and 15 of these (33.3%) presented SBP (Table 1). The mean age was 53.2 ± 12.3 years; 82.2% of the patients were men and 73.8% of them were Caucasian. About 60% of the individuals were classified as presenting Child-Pugh C and a mean MELD score of 19.5 ± 7.2. No individual was classified as Child-Pugh A, and only one presented MELD score ≤ 10. Besides SBP, we observed that 63.6% had jaundice, 35% hepatic encephalopathy and 37.1% UGIB. Only two individuals (6.5%) were using prophylactic antibiotics (norfloxacin), and none of them presented SBP.

**Table 1:** Comparative analysis of the clinical characteristics of 45 individuals with decompensated cirrhosis with ascites, in accordance with the presence of spontaneous bacterial peritonitis (SBP)

	Total	With SBP n = 15	Without SBP n = 30	P
Age (years)*	53.2 ± 12.3 (52.0)	49.7 ± 13.0 (46.0)	55.0 ± 11.8 (55.0)	0.175^†^
Male gender, n (%)	37 (82.2)	12 (80.0)	25 (83.3)	1.000^‡^
Caucasian, n (%)	31 (73.8)	10 (71.4)	21 (75.0)	1.000^‡^
Child-Pugh C, n (%)	16 (59.3)	8 (72.7)	8 (50.0)	0.427^‡^
MELD*	19.5 ± 7.2 (19)	22.2 ± 7.6 (20.0)	17.9 ± 6.7	0.067^†^
Complications				
SBP, n (%)	15 (33.3)	15 (100.0)	0 (0.0)	-
Jaundice, n (%)	28 (63.6)	11 (73.3)	17 (58.6)	0.336^‡^
Encephalopathy, n (%)	14 (35.0)	5 (41.7)	9 (32.1)	0.720^‡^
Upper gastrointestinal bleeding, n (%)	13 (37.1)	5 (41.7)	8 (34.8)	0.726^‡^
Etiology of the cirrhosis				
Hepatitis B, n (%)	4 (12.1)	1 (10.0)	3 (13.0)	1.000^‡^
Hepatitis C, n (%)	18 (47.4)	5 (38.5)	13 (52.0)	0.428^§^
Alcohol, n (%)	31 (73.8)	12 (85.7)	19 (67.9)	0.283^‡^
Comorbidities:				
Diabetes mellitus, n (%)	11 (37.9)	2 (20.0)	9 (47.4)	0.234^‡^
Hypertension, n (%)	12 (42.9)	5 (50.0)	7 (38.9)	0.698^‡^
Dyslipidemia, n (%)	1 (4.3)	0 (0.0)	1 (6.3)	1.000^‡^
AIDS, n (%)	3 (9.4)	11 (73.3)	17 (58.6)	0.336^§^

MELD = Model of End Stage Liver Disease

AIDS = acquired immunodeficiency syndrome

* Mean ± standard deviation (median)

† Student's t test

‡ Fisher's exact test

§ chi-square test

With regard to the etiology of the cirrhosis, it was observed that 46.7% showed alcoholism alone (from 73.8% patients with alcoholism), 20% HCV and alcoholism, 20% hepatitis C alone, 6.7% hepatitis B alone and 2.2% HBV and alcoholism. Table 2 describes the biochemical characteristics of the individuals with decompensated cirrhosis, and shows that 37.9% had DM, 42.9% SAH and 4.3% dyslipidemia. Acquired immunodeficiency syndrome was found in 9.4% of the individuals, in co-infection with HCV.

**Table 2:** Comparative analysis on the laboratory characteristics of 45 individuals with decompensated cirrhosis with ascites, in accordance with the presence of spontaneous bacterial peritonitis (SBP)

	Total	With SBP n = 15	Without SBP n = 30	P
Hemoglobin (g/dl)*	9.5 ± 2.4 (9.6)	9.1 ± 2.7 (9.1)	9.7 ± 2.3 (9.7)	0.385^†^
Leukocytes (/mm³)*	9.298.9 ± 6.768.7 (8.550.0)	11.921.3 ± 9.546.1 (8880.0)	7.987.7 ± 4.492.7 (8.000.0)	0.647^†^
Platelets (/mm³)*	134.266.7 ± 104.811.4 (109.000.0)	142.600.0 ± 102.083.2 (111.000.0)	130.100.0 ± 107.623.7 (76.500.0)	0.289^‡^
AST (U/l xULN)*	4.7 ± 7.7 (2.1)	7.8 ± 12.7 (2.2)	3.1 ± 2.2 (2.0)	0.804^‡^
ALT (U/l xULN)*	1.4 ± 1.6 (0.9)	1.7 ± 2.5 (0.6)	1.2 ± 0.8 (0.9)	0.144^‡^
ALP (U/l xULN)*	1.0 ± 0.8 (0.9)	1.1 ± 0.6 (1.0)	1.0 ± 0.9 (0.6)	0.126^‡^
GGT (U/l xULN)*	3.6 ± 3.5 (2.3)	3.2 ± 2.7 (2.4)	3.8 ± 3.8 (2.2)	0.559^†^
Albumin (g/dl)*	2.0 ± 0.6 (2.0)	1.9 ± 0.7 (1.9)	2.1 ± 0.6 (2.1)	0.466^†^
PA (%)*	43.5 ± 17.4 (42.8)	36.1 ± 16.0 (35.4)	47.1 ± 17.2 (44.5)	0.044^†^
INR*	1.7 ± 0.5 (1.6)	1.7 ± 0.6 (1.6)	1.6 ± 0.4 (1.6)	0.639^‡^
BRBT (mg/dl)*	5.5 ± 6.0 (2.8)	6.7 ± 5.9 (4.9)	4.8 ± 6.0 (2.0)	0.109^‡^
Creatinine (mg/dl)*	1.4 ± 0.7 (1.2)	1.6 ± 0.9 (1.4)	1.3 ± 0.5 (1.2)	0.303^‡^
Sodium (mEq/l)*	134.3 ± 5.9 (136.0)	133.1 ± 8.2 (133.0)	134.9 ± 4.3 (136.0)	0.245^‡^
Glucose (mg/dl)*	94.3 ± 23.4 (92.0)	91.4 ± 20.8 (91.0)	95.5 ± 24.8 (100.0)	0.524^‡^
Ascitic fluid analysis				
T proteins (g/dl)*	1.1 ± 0.7 (0.9)	1.2 ± 0.5 (1.2)	1.0 ± 0.8 (0.9)	0.153^‡^
Albumin (g/dl)*	0.3 ± 0.3 (0.3)	0.3 ± 0.2 (0.3)	0.4 ± 0.3 (0.3)	0.674^‡^
SAAG (g/dl)*	1.6 ± 0.6 (1.6)	1.3 ± 0.4 (1.2)	1.7 ± 0.6 (1.7)	0.045^‡^

AST = aspartate aminotransferase

ALT = alanine aminotransferase

ALP = alkaline phosphatase

GGT = gamma glutamyl transferase

xULN = times the upper limit of normality

PA = prothrombin activity

INR = international normalized ratio

BRBT = total bilirubin

T = total

SAAG = serum albumin ascitic gradient

* Mean ± standard deviation (median)

† Student's t test

‡ Mann-Whitney test

### Evaluation of the individuals included in the study, according to the presence of SBP

In the SBP group, the mean neutrophil count in ascitic fluid was 1,393.5 ± 1,115.1 cells/mm^3^, and three patients (21.4%) presented positive ascitic fluid cultures: two with *Klebsiella pneumoniae *and one with *Streptococcus sp*. The diagnostic paracentesis was performed, on average, after 3.6 ± 3.5 days of hospitalization.

In comparing the patient with SBP and the controls (Tables 1 and 2), it was observed that the patients with SBP presented lower mean PA (36.1 ± 16.0% versus 47.1 ± 17.2%; P = 0.044) and lower median SAAG (1.2 versus 1.7; P = 0.045). There was a tendency for the SBP group to present higher mean MELD scores (22.2 ± 7.6 versus 17.9 ± 6.7; P = 0.067). No difference was observed in relation to analyses on the following clinical and laboratory variables: age, gender, race, maximum axillary temperature, abdominal pain, diarrhea, Child-Pugh, jaundice, presence of encephalopathy, UGIB, death during hospitalization, hemoglobin, leukocytes, platelets, AST, ALT, GGT, ALP, albumin, total bilirubin, creatinine, sodium, glucose, proteins in the ascitic fluid and albumin in the ascitic fluid.

We observed a strong positive correlation between the neutrophil count in ascitic fluid and the serum leukocyte count (r = 0.501; P = 0.001). A negative correlation was also noted between the neutrophils count in ascitic fluid and PA (r = -0.385; P = 0.011). Significant correlations with hemoglobin, platelets, AST, ALT, ALP, GGT, serum albumin, INR, total bilirubin, creatinine, sodium, glucose, total proteins in the ascitic fluid, albumin in the ascitic fluid, SAAG and maximum axillary temperature were observed. 

## DISCUSSION

SBP has previously been described more frequently among males, at percentages ranging from 72.8% to 83.7%, which was similar to what was found in this study.^13^ ^-^ ^16^ Although the mean age among the individuals with SBP was somewhat lower than previously described by other authors (52.8 to 58.4 years),^13^ ^-^ ^16^ most patients were classified as Child-Pugh C (72.7%) with a high MELD score (22.2). Other authors have noted prevalences of Child-Pugh Class C ranging from 50.9% to 77.7%^14^ ^,^ ^16^ ^-^ ^18^ and mean MELD scores between 16.6 and 23.2, in line with what was found in the present study.^14^ ^,^ ^16^ ^,^ ^19^

With regard to the etiology of cirrhosis, Heo et al. reported that the greatest prevalence of cirrhosis was due to HBV (71.3%), followed by alcoholic cirrhosis (19.7%) and cirrhosis by HCV (6.4%),^16^ and these data are consistent with the high prevalence of HBV in Asia.^20^ ^-^ ^22^ In North America, Heidelbaugh et al. reported that alcohol was the main cause of cirrhosis (60-70%), followed by viral hepatitis (10%) and non-alcoholic fatty liver disease (10%).^23^ These findings were similar to those of a Brazilian study, conducted in Rio de Janeiro, which showed that in 39.9% of the cases, the etiology related to alcohol, 28.7% viruses, 11.9% mixed causes (alcohol and virus) and 14.7% a variety of causes.^18^ The prevalence of alcoholism in Brazil has been reported to range from 7.6% to 9.2%, which emphasizes the importance of this etiology as a cause of cirrhosis in our setting.^24^

Even though SBP is diagnosed as an ascitic liquid neutrophil count ≥ 250 cells/mm^3^, cellularities of up to 8,000 neutrophils per mm³ have been described.^25^ ^,^ ^26^ Despite the high cellularity found in this study, only 21.4% of the cases presented positive ascitic fluid cultures. In the literature, the prevalence has ranged from 12.6% and 68.4% of the cases.^13^ ^,^ ^15^ ^,^ ^18^ ^,^ ^25^ ^,^ ^27^ ^,^ ^28^

From reviewing the clinical and laboratory characteristics in relation to the presence of SBP, it was seen that there is controversy regarding the findings in the literature. The heterogeneity of the findings that have been correlated with the presence of SBP justifies indication of diagnostic paracentesis for all patients with decompensated cirrhosis with ascites who are admitted to hospital.^13^ Evans et al.^29^ assessed 427 patients with ascites and observed that 3.5% had SBP, but there were no significant differences in relation to serum albumin, serum bilirubin or INR between patients with and without SBP. Similarly to what was found in this study, it was reported that individuals with moderate to high MELD scores presented a substantially greater risk of development of SBP.^19^ ^,^ ^28^ At the same time, leukocytosis in peripheral blood can help to predict the appearance of SBP in asymptomatic patients with ascites.^14^ Other variables that have been described as predictors of SBP include C-reactive protein, erythrocyte sedimentation rate,^14^ UGIB and hypoalbuminemia.^18^

Figueiredo et al.^18^ evaluated 143 individuals with decompensated cirrhosis with ascites, among whom 20.3% presented a diagnosis of SBP. Among the variables analyzed, serum albumin (P < 0.001), C4 of ascitic fluid (P < 0.001) and UGIB in the previous week (P = 0.03) were identified as independent predictors for diagnosing SBP, and it was found that these combined variables could predict almost 97% of episodes of ascitic fluid infection. Kim et al.^30^ assessed 188 patients with cirrhosis and showed that, in comparison with patients with serum sodium ≥ 136 mmol/l, cirrhotic individuals with serum sodium concentration of ≤ 130 mmol/l presented significantly higher risk of development of SBP (33.3% versus 16.3%; P = 0.037) Guarner et al.^31^ evaluated 109 patients with ascites and cirrhosis, and discovered that 25.6% had developed SBP. Of the 20 variables evaluated in their study, 5 presented positive values that predicted the emergence of SBP: Child-Pugh score (P = 0.08); presence of encephalopathy (P = 0.06); serum bilirubin concentration (P = 0.007); total platelet count (P = 0.02); and total proteins in the ascitic fluid (P = 0.05). Only serum bilirubin count and platelet count presented independent correlations with the risk of SBP development. 

Such et al.^32^ evaluated 33 patients who had been hospitalized due to cirrhosis, of whom 21.2% had been diagnosed with SBP. Among the SBP patients, the complement C3 concentration in the ascitic fluid was significantly lower than in patients who had not developed ascitic fluid infection (9.0 ± 2.67 versus 18.26 ± 8.11; P < 0.01). The complement C4 concentration did not show any significant difference. Some serum markers were also indicated as predictors of SBP, namely: albumin (P < 0.05), bilirubin (P < 0.05) and PA (P < 0.05). Girón-González et al.^33^ studied 32 patients with cirrhosis and found that 20 of them had ascitic fluid infection. Their study showed that SBP was significantly associated with high serum levels of ICAM-I (P < 0.05), IL-8 (P < 0.01) and Gro-alpha (P < 0.01) and also with high levels of ICAM-I in the ascitic fluid (P < 0.05). A positive correlation was detected between PMN count in the ascitic fluid and IL-8 concentration (r = 0.65; P < 0.01). Coşkun et al.^34^ studied 50 individuals with cirrhosis, of whom 20% presented SBP. In their analysis, they demonstrated that nitrate levels were significantly higher in the patients with SBP than in the patients without SBP (282.4 ± 111.3 versus 186.4 ± 87.6; P < 0.05). In the same way, they showed that there were higher nitrate levels in the ascitic fluid of patients with SBP (302.4 ± 66 versus 135.4 ± 65.8; P < 0.001).

From comparing the factors associated with SBP in the present study with what has already been described in the literature, we perceived that these studies are discordant. Thus, each study found different variables to predict the appearance of SBP. Nonetheless, the present study, as well as the others, identified some variables as factors for the existence of SBP. These characteristics confirm that there is higher prevalence of SBP among individuals with advanced liver disease. 

The present study reinforces the recommendation that diagnostic paracentesis should be performed on hospital admission for all cirrhotic patients with ascites, in order to investigate the presence of SBP, even for patients admitted for reasons other than ascites, since no clinical characteristics other than the severity of liver disease can predict ascitic fluid infection.

## CONCLUSION

A few characteristics are associated with the presence of SBP, especially liver dysfunction (prothrombin activity), SAAG and peripheral blood leukocytes.
